# Efficient Deployment with Throughput Maximization for UAVs Communication Networks

**DOI:** 10.3390/s20226680

**Published:** 2020-11-22

**Authors:** Mohd Abuzar Sayeed, Rajesh Kumar, Vishal Sharma, Mohd Asim Sayeed

**Affiliations:** 1 Department of Computer Science and Engineering, Thapar Institute of Engineering and Technology, Patiala 147004, India; mohdabuzar@thapar.edu (M.A.S.); rakumar@thapar.edu (R.K.); 2 School of Electronics, Electrical Engineering and Computer Science (EEECS), Queen’s University Belfast (QUB), Belfast BT7 1NN, Northern Ireland, UK; 3 Department of Information and Technology, Babasaheb Bhimrao Ambedkar University, Vidya Vihar, Raebareli Road, Lucknow 226025, India; masayeed.rs@bbau.ac.in

**Keywords:** UAV, throughput, delay, packet loss, GNN, collaborative network, trajectory

## Abstract

The article presents a throughput maximization approach for UAV assisted ground networks. Throughput maximization involves minimizing delay and packet loss through UAV trajectory optimization, reinforcing the congested nodes and transmission channels. The aggressive reinforcement policy is achieved by characterizing nodes, links, and overall topology through delay, loss, throughput, and distance. A position-aware graph neural network (GNN) is used for characterization, prediction, and dynamic UAV trajectory enhancement. To establish correctness, the proposed approach is validated against optimized link state routing (OLSR) driven UAV assisted ground networks. The proposed approach considerably outperforms the classical approach by demonstrating significant gains in throughput and packet delivery ratio with notable decrements in delay and packet loss. The performance analysis of the proposed approach against software-defined UAVs (U-S) and UAVs as base stations (U-B) verifies the consistency and gains in average throughput while minimizing delay and packet loss. The scalability test of the proposed approach is performed by varying data rates and the number of UAVs.

## 1. Introduction

The flexible applicability and ability of unmanned aerial vehicles towards fast, cost-effective, and temporary deployments has opened a broad spectrum of possibilities for future wireless technologies. UAVs can be deployed in virtually every scenario, from cellular base stations to disaster relief and response vehicles. Aerial networks have a line of sight (LoS) advantage and the high altitude deployment itself is a major factor behind improved coverage. UAV-assisted ground networks have already taken cooperative search, acquisition, and tracking (CSAT) to new dimensions. Cooperative ad hoc network formations have led to major advances in civilian and military applications. Aerial and ground communication networks, when laced in conjunction, facilitate an efficient entourage for supervision, catastrophe reassurance, observation, investigation, and supplementary applications [1,2,3,4,5].

The race of evolution in wireless technology has graduated the use of UAVs from the military to the Civilian Concepts of Operations (CONOPS). Enhanced coverage, throughput maximization, sustainable operating costs, and ease of deployment constitute the fundamental obligations towards UAV-CONOPS. CBRN (chemical, biological, radiological, and nuclear reconnaissance)-CONOPS are focused on hazard containment and mitigation [6,7,8,9]. UAV collaborative networks have paved for a level playing field for data dissemination, broadcast/multicast communications. The design goals of multi-UAV collaborative networks have a major focus on altitude and position optimization and spatial density for better coverage.

Generally, UAVs act as aerial base stations to support ground communications, be it cellular, sensor, relief, and response or data dissemination and hence hovering altitude or geographical positioning along the (x,y) axis can be jointly or separately optimized to achieve varying levels of performance gains [10,11,12,13]. UAVs can also serve as a middle man for coverage enhancement and boosting capacity [14,15,16]. With the emergence of 4G LTE and 5G communication technologies, cost-effective coverage enhancement has been a topic of interest. The issue can be easily resolved by employing UAVs as mobile base stations or temporary relays [17,18]. The UAV hovering and optimal placement can boost overall capacity and throughput of the Internet of Things (IoT) communications [19,20].

The unexplored potential of UAV assisted networks brings certain challenges alongside. Trajectory design, resource allocation, channel allocation, and tradeoffs between throughput and delays are a few identified challenges towards maximized data rates in multi-UAV collaborative deployments [21]. UAV-assisted ground networks are due to gain significantly from considering and exploiting the flexible mobility characteristics of aerial nodes. UAV nodes are highly maneuverable and can provide greater opportunities for LoS channel availability and better capacity with on-demand trajectory modifications [22,23]. The on-demand availability of UAV mobility instead of predetermined paths can alleviate the restrictions incurring from high latency and transmission losses.

Dynamic control and state estimation of the aerial network are driving forces behind the optimized UAV trajectory. The proposed approach models throughput maximization and capacity enhancement of the multi-UAV assisted ground networks as a trajectory optimization paradigm and considers UAV state as the criteria for UAV re-purposing. UAV re-purposing is effectively a technique where available less congested UAVs are directed towards geographical sectors with high latency and packet loss rates. The state of the aerial nodes is determined iteratively using graph neural networks (GNN). GNN architectures use iterative message passing to produce a cumulative state information vector employing aggregation schemes at each level [24,25,26,27]. The proposed GNN’s node embedding incorporates UAV’s position concerning sector anchor in a 3D area and node features, including throughput, latency, and packet loss. Incorporating UAV positioning is important for feature state calculation as two aerial nodes with the same characteristics are indistinguishable without geo-positioning.

The majority of approaches following multi-UAV-assisted throughput maximization have studied trajectory optimization as a function of underlying ground nodes and application scenarios. The proposed approach performs iterative learning at both local and global topological levels and considers application scenario independent ground nodes, which makes it more reactive and adaptable to sudden changes in geography, node failures, and bottlenecks as compared to the mathematical optimization techniques proposed in the literature. Trajectory and throughput optimization require tracking multiple aerial and ground nodes. The techniques proposed in the literature have traditionally investigated linear tracking in a plane. The simplification reduces the throughput maximization into an optimization problem but does not address the aerial node tracking when targets move in a 3D plane. While multi-UAV trajectory optimization generally focuses on aerial nodes moving in the 2D front parallel plane, this is an atypically simple, special case. The proposed approach considers aerial nodes moving continuously in all three dimensions. Moreover, 2D tracking suffers a steep performance decline when speed and distances increase, as overall mapping accuracy is always higher in 3D than in 2D. The proposed approach employs separation and dimensionality to provide more than additive improvements as the aerial nodes packed closely together in a 2D front parallel perspective can be far apart if altitude is considered, and can be mapped accurately in a 3D geography. Moreover, the mapping accuracy improves when aerial nodes are separated by different altitude planes.

In this paper, we present a multi-UAV assisted ground network, where UAVs are deployed as transceivers as well as base stations in a given 3D area. A dynamically reconfigurable topology is presented where state information of aerial nodes is generated and updated periodically to keep track of congestions and declining throughput. The proposed approach aims at the throughput maximization of UAV-assisted ground networks. The contributions of this paper are summarized as follows.

1. Effectively monitoring the state of aerial nodes and aerial topology for traffic patterns and link congestion. Modeling aerial nodes and associated links for traffic characterizations, delay, loss, and throughput to estimate the topological re-configurations and capacity predictions.
2. Pushing data rates close to the throughput upper bound of UAV-assisted ground networks through aerial node re-purposing, reinforcing burdened nodes, and links. Throughput is maximized by pushing data through new routes created by adjusting UAV positions, which in turn minimizes delays and loss.
3. The proposed approach can act as an overlay and can accommodate any kind of UAV-assisted network configuration. It is feasible to scale the approach to accommodate any number of ground and aerial nodes, given that the data rate is evenly matched.

The rest of the paper is organized as follows: Section 2 presents the most recent developments in the field of UAV trajectory modeling and throughput maximization. Section 3 covers the foundations of path problems in UAV networks and also discusses why Graph Neural Network (GNN) is more suited as a solution than traditional neural network architectures. Section 4 provides a theoretical and mathematical description of the proposed approach. For performance analysis, the proposed approach is compared against software-defined UAVs (U-S) and UAVs as base stations (U-B) inspired by the configurations in [28,29], respectively. Section 5 also presents proof of correctness and scalability analysis of the proposed approach. Finally, Section 6 concludes the paper.

## 2. Related Works

Wu et al. [30] proposed a multi-UAV assisted ground wireless communication network, where UAVs serve as mobile aerial base stations facilitating ground connectivity. To guarantee fair performance among the ground nodes, minimum throughput criteria are maximized for the downlink communications. The approach is directed towards optimized scheduling of multi-user communication and association alongside UAV trajectory. The availability of UAV connectivity as a result of geographical dynamics is achieved by means of a trajectory installation algorithm based on circular trajectory and circle packing methods. In order to achieve minimum average data rates, an iterative algorithm is proposed. The technique uses an interleaved trajectory and power optimization with each iteration. Xie et al. [31] studied UAV coordinated (UAV) wireless powered communication network (WPCN), where UAVs are employed as mobile access points to serve ground nodes. The UAV uses radio frequency (RF) wireless power transfer (WPT) for downlink, while ground nodes use harvested RF energy for uplink. The proposed technique aims at maximizing the minimum throughput criteria by achieving an effective trajectory design and optimized resource allocation.

Lin and Saripalli [32] presented a route planning algorithm for UAV collision avoidance. The technique features three variants of closed-loop rapidly-exploring random tree algorithm: trajectory generation, intermediate way-point utilization, and prediction of obstacles and collision. Qian et al. [33] proposed user association optimization for UAV enabled mobile edge computing (MEC) applications. UAVs are employed as edge computing servers to allow user equipment and ground nodes to offload tasks. Optimized UAV trajectory, user association, and uplink power of the ground nodes facilitate maximized offload bit rates of ground nodes. An optimized UAV trajectory for minimized localization errors is presented in [34]. The authors have studied that terrestrial node localization is cost-effective, fast, and more accurate when aerial nodes are employed and proposed a framework for node localization errors in dense urban environments. Altitude, flight time, the density of waypoints, and distance characteristics are considered to solve the constrained localization problem.

Sayeed and Kumar [35] proposed an intelligent selection of waypoints based on attraction factor, for capacity enhancement in multi-UAV guided WSNs. The transmission density-based attraction factor is calculated to achieve maximum throughput and coverage with minimized aerial node deployment. Genetic algorithm-based UAV trajectory generation is discussed in [36]. The proposed approach targets minimum fuel requirements and low altitude to avoid detection. A SDN-based mobility mechanism for UAV-assisted ground networks is presented in [37]. The authors studied that to maintain an efficient ground to air collaboration, securing the networked environment is of paramount importance. The proposed technique addresses coverage requirements by a density-based selection of UAV waypoints. The SDN controller sits on top of the overall network and issues security certificates to guard against faulty nodes, selfish nodes, intrusions, and malicious attackers. Dynamic trajectory generation and modification are discussed in [38].

Mardani et al. [39] proposed an offline algorithm to solve the optimal pathfinding problem in two-dimensional space, in order to guarantee maximized throughput criterion for cellular video streaming. The technique implements two variants of the classical A* algorithm directed towards optimizing distance and UAV throughput. The proposed technique considers energy, the state of wind, and the path post smoothing as the main players over which the problem is formulated. The algorithm optimizes video streaming quality while preserving the overall system energy. Moreover, the proposed solution is integrated into the real world by implementing the algorithms into the QGroundControl (QGC) control station.

Ant colony optimization (ACO)-based UAV trajectory optimization for enhanced coverage and collision avoidance is discussed in [40]. ACO-based trajectory optimization for search time minimization is presented in [41]. ACO-based coordinated path planning for multi-UAV networks under threat conditions is discussed in [42]. Three-dimensional path planning for robots to avoid local maxima is elaborated in [43]. Parallel genetic algorithm-based multi-UAV path and trajectory is discussed in [44]. Evolutionary trajectory planner for unrealistic multi UAV scenarios is discussed in [45]. Genetic algorithm-based age optimal trajectory planning is implemented in [46].

Wu and Zhang [47] stated that UAV mobility is limited by the delay requirements of the overall network, and aimed towards performance gains achieved via delay constrained communications. The UAVs act as a mobile base station for the ground nodes and the minimum rate ratio is used to manipulate the delay limited data traffic. The approach aims at optimizing global minimum average throughput by means of optimized UAV trajectories and OFDMA resource allocations. The proposed technique successfully translates the throughput-delay tradeoff of UAV networks and a trajectory installation mechanism driven by a simple circular trajectory is proposed. Wu et al. [48] discussed UAV-enabled wireless networks with UAVs serving as mobile base stations. The technique aims at guaranteeing fair and equal performance to the underlying ground nodes by means of optimizing minimum throughput. Global throughput optimization is achieved by the joint optimization of trajectory and multiuser communication scheduling.

Ahmed et al. [49] presented an energy-efficient UAV trajectory modelling technique. The approach aims at maximizing the network throughput while optimizing the required UAV propulsion energy, or simply put, UAV energy. Two-dimensional geometry for UAV deployment is considered, as UAV is assumed not to change its altitude in order to avoid collisions. Initially, optimal trajectory, transmit power, UAV speed, and UAV scheduling is considered for throughput optimization. In the second phase, UAV propulsion energy is considered as a function of UAV trajectory and speed. The UAV energy is maximized in terms of energy consumed per bit of information transmitted between UAV and ground nodes given a continuous flight.

Liu and Zhu [50] presented an effective transmission policy for UAV-assisted WSN networks and trajectory optimization for aerial networks. The preplanned UAV trajectory focuses on energy-efficient data transmissions and time-bound servicing of the ground nodes. A dynamic programming solution is presented for optimizing the transmission policy. Then, recursive random search is employed for developing a preplanned UAV route over the established dynamic transmission policy. Tang et al. [51] proposed a UAV trajectory planning algorithm that optimizes the UAV path by minimizing the UAV deviations. The authors have studied that natural constraints always force UAV to deflect from its preplanned course. The algorithm employs minimum snap trajectory methods to construct triangles over the pre-planned trajectory coordinates and then applies corridor constraints to minimize UAV deviations.

Li et al. [52] studied resource allocation in UAV powered networks, where UAV acts as a base station or access point to service ground user nodes. The proposed technique aims at communication throughput maximization through generating optimal UAV trajectory and carrier allocation policy, given the minimum data rate required to service each ground node. Ouyang et al. [53] considered an aerial communication system where laser transmitters are employed to charge the in-flight UAVs and the UAV-harvested energy is used to facilitate aerial-ground communications. The framework restricts the UAVs from exceeding the energy consumption to that of harvested energy. The proposed technique maximizes the downlink throughput by joint optimization of trajectory and transmission power. Bulut and Guevenc [54] considered UAV trajectory optimization where an in-flight UAV does not lose cellular connection from the ground base station.

Tang et al. [55] proposed a multi-agent deep Q learning (DQL) strategy to optimize UAV trajectory and resource allocation to maximize throughput in multi-UAV guided wireless powered communication networks (WPCNs). The mobile UAV base stations are used to charge the underlying internet of things (IoT) devices; in return, the IoT devices use this energy for data transmission between the device and aerial nodes. The proposed technique optimizes the three dimensional UAV path and channel resources with the added constraints of UAV speed and transmission power of IoT uplink. Rahman et al. [28] defined UAV trajectory as the major factor affecting the overall network capacity serving the ground nodes. The proposed technique aims at throughput maximization in disaster areas. The UAV enabled a software-defined disaster area network to monitor the network topology at all times and maintains flows. The data rate of overall topology is managed to provide maximized throughput. Sivalingam [29] proposed that deploying multiple aerial nodes working as base stations can help uplift the coverage problem and maximize throughput. The authors have proposed an algorithm for determining locations within the pool of predetermined locations.

Xie et al. [56] considered UAV-driven two-user interface channels that are used to charge two on-ground IoT devices. The charged devices in turn transmit information to the aerial nodes. For the UAV driven WPCNs, the UAV trajectories are designed in a way that they boost the overall wireless power transfer alongside canceling the channel interference. The proposed technique optimizes the uplink throughput of the IoT devices by joint optimization of UAV path trajectory and resource allocation of uplinks and downlinks. The algorithm is constrained by UAV speed, UAV collision avoidance, and energy neutrality of IoT devices. Liu et al. [57] proposed a relay technique that takes advantage of UAV mobility and performance. The proposed technique aims at throughput maximization by optimizing UAV trajectory and transmission power.

Xu et al. [58] discussed multi-UAV-enabled wireless communication where aerial nodes are deployed as a sink for the energy-restricted/-constrained ground terminals. The technique also incorporates a security mechanism for UAV collision avoidance. The minimum throughput of the ground nodes is optimized to dissipate fairness among the ground nodes. The optimal minimum throughput is achieved by optimizing communication scheduling, energy, and UAV path trajectories.

Jiang et al. [59] employed UAVs for data transmission between disconnected ground nodes. The proposed technique considers the changes induced into the transmission channel as a result of UAV movements. The overall network throughput is maximized by optimizing transceiver power allocations and UAV trajectory. Zeng and Zhang [60] introduced a circular UAV trajectory to enhance the energy efficiency of the network by optimizing the path radius and flight speed. A propulsion energy model is proposed, considering flight speed, direction, and acceleration as parameters. The technique aims at optimizing overall network throughput and energy through optimized trajectories.

Zeng et al. [61] aimed to minimize the energy consumption of UAV-enabled ground communication networks. The technique optimizes the UAV positioning and trajectory and develops over a traveling salesman problem. The total energy consumption requirement is satisfied alongside the minimum throughput requirements of the ground nodes utilizing UAV trajectory optimization and time slot allocation for ground nodes. Zhang et al. [62] employed multi-hop UAV networks for data dissemination between source and destination nodes. The proposed technique maximizes end-to-end throughput via trajectory and transmission power optimization. Wu et al. [63] proposed throughput maximization in multi-UAV powered WPCNs. The ground nodes use harvested energy from mobile wireless energy transfer for uplink and downlink. Minimum throughput is maximized by optimizing UAV trajectory and resource allocation.

Cheng et al. [64] proposed UAV trajectory optimization for data offloading at base stations. The technique maximizes the combined throughput of the edge users through optimized UAV trajectories. Hua et al. [65] proposed maximized system capacity by alternatively optimizing trajectory, end-user scheduling, and transmit power available to the end-users. Zeng et al. [66] studied data dissemination in multi UAV-enabled multi-cast ground networks. The proposed technique creates an optimal trajectory design that ensures minimum data dissemination time and guarantees a high probability of the ground node reception.

## 3. Network Model

**Figure 1:**
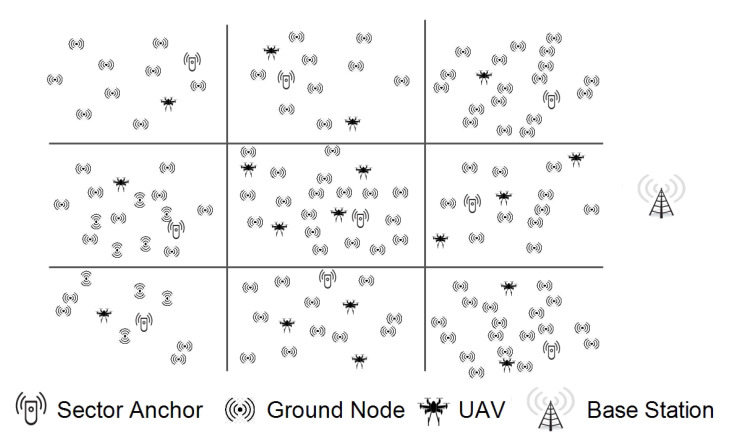
Proposed approach: Network model.

UAV networks are complex design paradigms banking on effective trajectory selections, situation awareness, and communication in conjunction with dynamic network reassessment and load characterization. Without featuring a dynamic trajectory modification in accordance with the iterative situational-updates, the overall system performance declines, given sufficient ground and aerial resources. The proposed approach focuses on throughput maximization in multi-UAV assisted ground networks. The technique employs UAV mapping and trajectory optimization using UAV re-purposing to minimize delays and packet loss and maximize throughput. The network comprises aerial and ground nodes. The UAVs act as both transceiver and base for the underlying ground network. Aerial nodes initially follow a predetermined path through the sectors. A sector is a 3D volumetrically equal division of the geographical topology. Sector anchor is a set of random points in a divided 3D plane such that each division has at least one anchor. A single UAV topology can be classified into two categories within the same temporal instance. Local topology defines a node’s attributes concerning its immediate neighbors. The global topology defines the UAV positioning concerning the overall geographical deployment. Figure 1 details the complete network layout. The topological characterization reveals the relationship dynamics of the UAV-assisted ground network deployment. To perform trajectory optimization and UAV re-purposing, it is important to map aerial and ground nodes to particular sectors. Unless intervened, UAVs fly autonomously over a predetermined trajectory with constant velocity.

Each aerial and ground node is mapped to its corresponding sector in accordance with the local network layout. The corresponding sector anchors for each sub-area are marked. The sector anchor can be adjusted to the center of the node cluster within its specific sub-area. The connected mesh of aerial nodes serves as the initial network map or graph. As the network progresses, with continued iterative transmissions, each node calculates its own set of features and link characteristics of the adjoining nodes. Every time the network gets up and running, graph depended shortest path algorithms are used to initialize the network. The issue with the shortest path or multipath approaches is that they do not take into account the distance and latency of the links while updating paths during their initialization phase. The problems associated with the absence of steady and dynamic learning of network patterns cause complete re-initializations of the paths around the node where the link is broken or congested.

Collaborative networks rely on traditional multi-hopping techniques for data dissemination. The data path from source to destination is calculated employing graph algorithms (example: Dijkstra’s shortest path algorithm or Bellman–Ford algorithm). The network itself forms a dynamic graph with temporary connections. The shortest and most efficient link from *i* to *j* can be a single shortest path; however, it suffers from delay and packet loss due to congestion. The tremendous decline in overall network performance and inefficient data transmissions result from slow reaction and path reconstruction, link failures originating from the dynamic topological arrangements, flooding, network clogging, and higher latency towards alternate route calculation. Moreover, the traffic originating from one subsection of the network will start sharing the paths when destined to another subsection, further increasing the latency and impacting overall throughput.

The solution can be found in multi-path techniques for providing alternate paths and reduced latency, but it further increases the complexity and generates overheads for calculating and maintaining multiple paths from source to destination. Maintaining all the multiple paths is generally not required for multi hopping transmissions and can be avoided by employing efficient trajectory optimization, thus boosting overall network performance. The article presents a solution that provides a multipath arrangement for congested links and burdened nodes through UAV re-purposing. It starts by calculating node and their link states, including the state of its neighbors, and updates the node parameters. If an additional path is required by the network in an area, the UAV positioning is adjusted and new paths are dynamically arranged. The UAV is re-positioned according to the criteria which maintain old links alongside the new.

**Figure 2:**
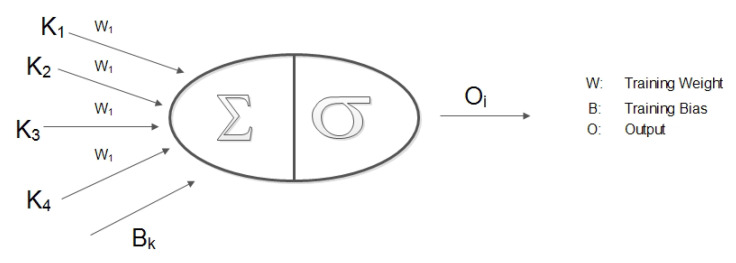
Graph neural network: Output generation.

**Figure 3:**
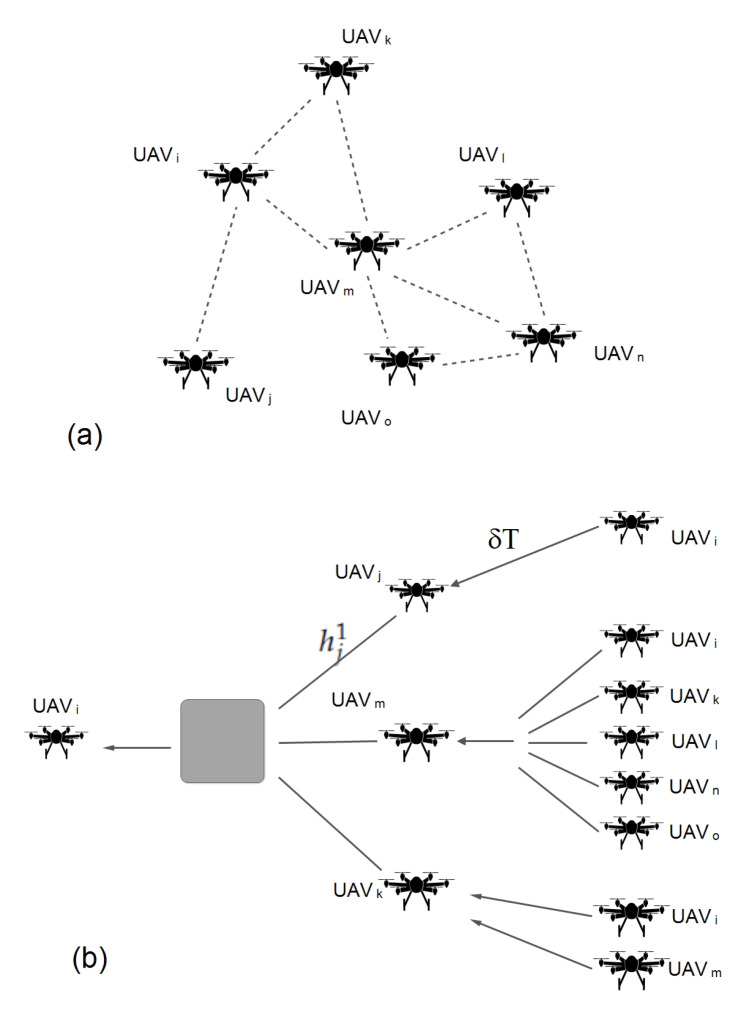
(**a**) Topological relationship of *i*th UAV. (**b**) Aggregate state definition of *i*th UAV.

GNN has a specific ability to acknowledge the natural order of nodes in a graph, i.e., no particular order but traversing the nodes in all possible orders. Traditional neural models process the patterns in a stacked specific order which makes them less suited towards the dynamic nature of topology and fast movement of the aerial nodes. In a network graph, a connection means the state of the node which itself is dependent on the state of neighboring nodes and links. Traditional neural models account for this interconnected dependency, as a feature of the node itself, whereas GNN performs propagation guided by the graph structure instead of using it as part of features. GNN can retain states up to arbitrary lengths, thereby better capturing the graph dependence of states via transmission and movement characteristics. The GNN operations, in the proposed approach, can be summarized as: calculate node state, propagate node state along the edges of the graph and re-calculate node states according to the received updates. The functionality of GNN in coordination with the proposed approach is detailed in Section 4. Equation (14) and Figure 2 elaborates on the operational GNN feed-forward network and state output calculation. Figure 3 shows how UAVs propagate the feature vector to the neighboring UAVs. The recipient aerial nodes append their feature vectors according to the received updated vector from the neighbors.

**Figure 4:**
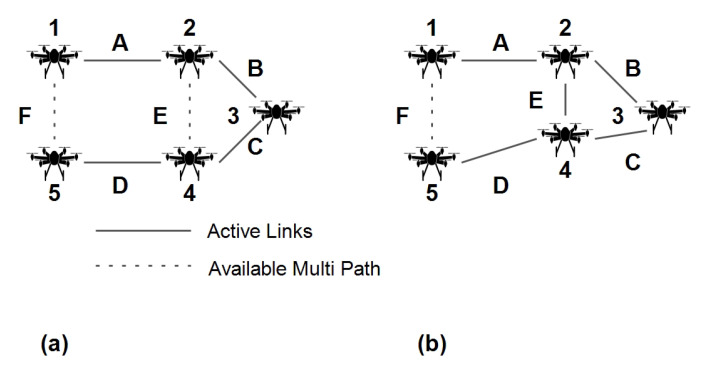
Proposed approach: (**a**) Network graph with congested link B (**b**) UAV re-purposed to activate link E alongside B.

Example: The UAV re-positioning can facilitate alternative paths where a node/link is broken/ congested. The node state is used to decide upon moving in another UAV with desirable current state characteristics. The path in a given set of UAV links is denoted by ABCD (Figure 4). ABCD are weights whose values depend upon the feature vector. The GNN can learn this graph alongside node features and link states. During steady network operation, suppose link *B* becomes congested. A graph neural network will present this abrupt change in behavior in $O_{i}$ Equation (11). The proposed approach now estimates an aerial node close to $UAV_{1}$ or $UAV_{2}$ and re-purposes it to send packets from $UAV_{1}$ to $UAV_{5}$ using link *E*. The complete state estimation and UAV re-purposing techniques are presented in Section 4.

## 4. Proposed Approach

The proposed approach aims at pushing the maximum data rate of UAV-assisted ground networks towards the throughput upper bound of-UAV assisted ground network deployment. Maximum data rates are achieved by minimizing the overall latency and packet loss. UAV re-purposing achieves minimal packet loss and delays by allowing dynamic topological changes considering the state of the UAV, state of the neighboring nodes, and the overall state of the sector. Individual UAV feature vectors are used to collaboratively determine the state of an aerial node concerning its neighbors and load characteristics. The initial feature vector is defined over the node’s latency, loss, observed throughput, and distance from the sector anchor. GNN aggregation is used iteratively to determine each UAV state with respect to its one-hop neighbors and so on. The dynamic and continuous node assessment keeps track of each aerial node and its network statistics. The overall sector state is assessed against the UAV states, and congested nodes (high latency and packet drop) are served using re-purposed UAVs.

Network latency is a numerical measure of delay and is described as the time it takes for a signal to propagate across the network connection, towards its destination. The latency of a UAV-assisted ground network is defined as Equation (1):

(1) $$L_{w}=l_{r}+l_{q}+\left(l_{t}\times h_{c}\right)+l_{p}+l_{c},$$

where $L_{w}$ is the latency of a wireless link, $l_{r}$ is the router traversal latency, $l_{q}$ is queuing delay and is defined as the amount of time that data packet spends in the queue before transmission, $l_{t}$ is transmission delay and is a measure of time, it takes to place an entire packet on the transmission channel. Transmission delay is the ratio between packet size and transmission rate. $h_{c}$ is hop count, $l_{p}$ is the propagation delay, or the signal propagation time across the transmission channel. Propagation delay is the ratio between the node displacement and speed of the communication channel. Connection delay ($l_{c}$) is the time it takes to establish the connection between aerial and ground nodes.

Packet loss rate or packet loss probability of a UAV-assisted ground network is defined as Equation (2):

(2) $$L_{p}=\frac{Tx_{p}-Rx_{p}}{Tx_{p}},$$

where $L_{p}$ is the packet loss ratio, $Tx_{p}$ is the actual number of packets transmitted and $Rx_{p}$ defines the packets received.

Network throughput is the rate of packet delivery along with a communication network. Mathis et al. [67] proposed the upper bound on the data transfer rate over a communication channel. The throughput upper bound $Th_{max}$ for a UAV-assisted ground network is defined as Equation (3):

(3) $$Th_{max}\leq MSS\times \frac{1}{I_{let}}\times \frac{1}{I_{del}},$$

where *MSS* is the maximum packet size.

(4) $$I_{let}=\frac{1}{n}\sum_{i=1}^{n}L_{w_{i}}=\frac{1}{n}\sum_{i=1}^{n}l_{r_{i}}+l_{q_{i}}+\left(l_{t_{i}}\times h_{c_{i}}\right)+l_{p_{i}}+l_{c_{i}},$$

(5) $$I_{del}=\frac{1}{n}\sum_{i=1}^{n}L_{p_{i}}=\frac{1}{n}\sum_{i=1}^{n}\frac{Tx_{p_{i}}-Rx_{p_{i}}}{Tx_{p_{i}}},$$

and

(6) $$I_{del}=\left\{\begin{array}{cc} 1 & \;\mathrm{if}\;Tx_{p_{i}}=Rx_{p_{i}}\\ \frac{1}{\frac{1}{n}\sum_{i=1}^{n}L_{p_{i}}} & \;\mathrm{otherwise} \end{array}\right.$$

In order to achieve the maximum data rates in UAV-assisted ground networks ($Th_{maxw}$), the proposed approach minimizes the $I_{let}$ Equation (4) and $I_{del}$ Equation (5), which in turn can be minimized by minimizing the $L_{w}$ Equation (1) and $L_{p}$ Equation (2). The goal of the proposed approach is to achieve $Th_{maxw}$ values close to and approaching $Th_{max}$ Equation (7).

(7) $$Th_{maxw\to max}\propto min\left(L_{w},L_{p}\right)$$

An instance of a multi-UAV-assisted ground network constitutes *n* number of UAV nodes in a 3D plane participating in a collaborative network formation. The dynamic movement of nodes in (x,y,z) plane imposes significant difficulty in network reconfiguration for optimal channel utilization and data rate maximization. A feature vector is designed to represent the link wise network state which incorporates latency and congestion over each link. The feature vector δT at the *i*th node is defined as Equation (8):

(8) $$\delta T⟵\{L_{w_{i}},L_{p_{i}},Th_{0_{i}},d_{i}\},$$

where $Th_{0_{i}}$ is the observed data rate at *i*th node and d is node’s displacement from the sector anchor. The $d_{i}$ is the Mahalanobis distance [68] calculated between node positions and sector anchor. $d_{i}$ is defined as Equation (9):

(9) $$d_{i}=\sqrt{\left(y-\mu \right)C^{-1}{\left(y-\mu \right)}^{\prime }},$$

where *y* is the set of anchor points, μ is the mean of UAV coordinates and *C* is the covariance matrix.

UAVs hover with random way-point selection or any other predefined mobility, but are ready to change course and follow a newly set path as dictated by the proposed trajectory optimization technique. The multi-hop UAVs will change their adjacency relationships more frequently than the single hop nodes. The local and global arrangements can be represented as graph $G(U_{\left(x,y,z\right)},A)$ and adjacent matrix $A_{\left(i,j\right)}$ Equation (10).

(10) $$A\left(i,j\right)=\left\{\begin{array}{cc} 1 & \;\mathrm{if}\;uav_{i}\;\&\;uav_{j}\;\mathrm{are}\;\sin\mathrm{gle}\;\mathrm{hop}\;\mathrm{nodes}\\ \;0 & \;\mathrm{if}\;uav_{i}\;\&\;uav_{j}\;\mathrm{are}\;\mathrm{multi}\;\mathrm{hop}\;\mathrm{nodes} \end{array}\right.$$

At any reference interval, the exact state of the aerial network must be accounted for to facilitate the efficient positioning of the relay nodes. The state of the aerial network can be heuristically defined using graph neural network [69]. The expected behavior of the node is required to adjust the positioning of the nodes in advance to push the overall network capacity towards $Th_{maxw}$. With the graph neural network, the *i*th UAV’s state $O_{i}$ is given by Equation (11). This state is defined over the current state of the UAV node and its neighboring UAVs, presenting the neural network output considering the UAV’s feature vector and input vectors received from the neighboring UAVs. The number of layers considered in the proposed model is equal to the graph levels formed by UAV nodes in a sector. The arrangement of layers according to UAV topology is described by Figure 3. The UAV node’s state vector $h_{i}$ concerning neighboring UAVs is described by Equations (11) and (12):

(11) $$O_{i}=\rho_{w}\left(h_{i},\delta T_{i}\right),$$

(12) $$h_{i}=\Upsilon_{w}\left(\delta T_{i},A_{\left[i\right]},h_{ne[i]},\delta T_{ne[i]}\right),$$

where $\delta T_{i}$ is the feature vector, $A_{\left[i\right]}$ is the adjacency matrix for *i*th node. $h_{ne[i]}$ defines state of *i*th UAV’s neighbours. $\delta T_{ne[i]}$ define features of the neighbouring UAVs.

The state *h* of an aerial node at the 0th layer is given by Equation (13). The 0th layer UAV treats its feature vector as its state. The 0th layer is the initial layer from the disjoint graph considered for UAV state calculation.

(13) $$h_{i}^{0}=\delta T_{i}.$$

At *k*th layer, the state of *i*th node can be defined as Equation (14):

(14) $$h_{i}^{k}=\sigma \left(W_{k}Ag,B_{k}h_{i}^{k-1}\right),$$

where $W_{k}$ and $B_{k}$ are weight and bias respectively, used for training the neural network at *k*th layer and Ag is the aggregate function Equation (15) with parameters α, β, γ and δ Equation (16). Algorithm 1 presents the UAV state calculation process.

(15) $$Ag=\sum_{i=0}^{n}\left(\alpha L_{w_{i}}+\beta L_{p_{i}}+\gamma Th_{0_{i}}+\delta d_{i}\right),$$

where *n* is the number of nodes in a divided sector.

The proposed approach considers a maximum of three hop neighbors’ states. The actual UAV topology over geography and the definition of UAV states derived from the geographical layout are presented in Figure 3.

(16) α+β+γ+δ=1.

$UAV_{i}$ will have a state corresponding to its latency, packet drop, instantaneous throughput, and distance from sector anchor, given by Equation (11). UAV re-purposing is required to ensure consistent and improved data rates if the throughput of a sector $Z_{rate}$ Equations (17) and (18) falls below the expected throughput. The expected rate $Z_{exp}$ is proportional to the channel capacity $C_{s}$ of the sector, such that

(17) $$Z_{rate}\propto \frac{1}{\sum_{i}^{n}O_{i}},$$

(18) $$Z_{rate}<Z_{exp}|Z_{exp}=\frac{C_{s}}{2}.$$

The distance $d_{ai}$ between a sector anchor $A_{i}$$\left(A_{ix},A_{iy},A_{iz}\right)$ and an aerial node $UAV_{i}$$\left(U_{ix},U_{iy},U_{iz}\right)$ in a w-dimensional space is given by Equation (21).

**Figure 5:**
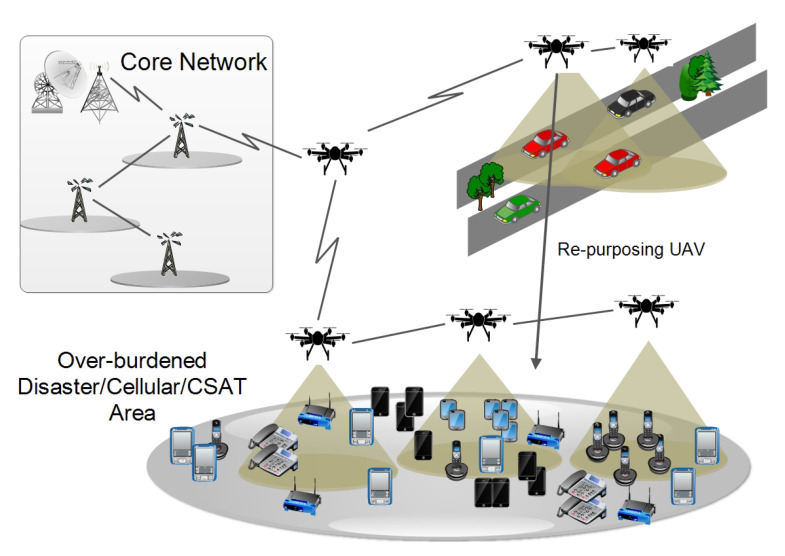
An illustration of state-based UAV re-purposing.

The candidate solution for UAV re-purposing is assigned according to Equation (19). UAV is re-purposed to a designated overburdened high latency node such that its distance from the sector anchor $d_{ai}$ and current state $O_{i}$ are minima. Algorithm 2 elaborates on the UAV trajectory optimization and UAV re-purposing process. Figure 5 details the UAV re-purposing scenario.

(19) $$min(O_{i},d_{ai}).$$

Tangent of the *i*th UAV towards the burdened node is used to set the re-purpose direction and the minimum required movement $d_{m}$ is assigned according to the prorogation length $Sp_{i}$ of the UAV signals Equation (20).

(20) $$d_{m}=\frac{Sp_{i}}{2}.$$

If no UAV nodes are available for re-purposing, a directly connected UAV with min current state $O_{i}$ value is considered.

(21) $$d_{ai}=\sqrt{{\left(A_{ix}-U_{ix}\right)}^{2}+{\left(A_{iy}-U_{iy}\right)}^{2}+{\left(A_{iz}-U_{iz}\right)}^{2}}=\sqrt{\sum_{r=1}^{w}{\left(A_{ir}-U_{ir}\right)}^{2}}.$$

**Figure 6:**
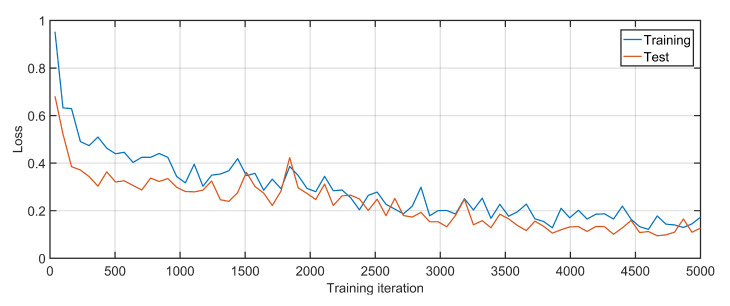
Loss: Training and testing.

To estimate the cost and complexity of the proposed solution, the initial training of the algorithm is performed using 10 formations of UAV networks, and the GNN is trained over 5000 iterations, followed by re-testing using 5 formations of the UAV network. Figure 6 gives training and testing losses over each iteration, where 1000 iterations take 4 min to complete approximately. The results suggest that the loss is quickly reduced and the GNN is able to correctly predict the node states.
**Algorithm 1:** UAV state identification
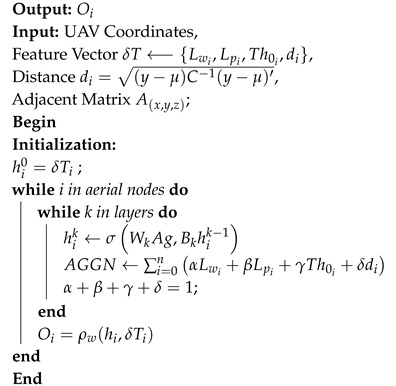

**Algorithm 2:** UAV re-purposing
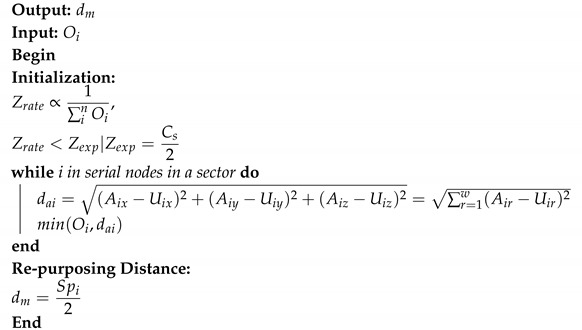


## 5. Results and Discussion

To evaluate the proposed approach, simulations are carried using NS-3 (Python bindings) with 50–100 UAVs and an area of 2000×2000 m. Comparisons are made on grounds of achieved throughput, delay, packet loss, jitter, and packet delivery ratio. To demonstrate the accuracy and correctness, the proposed approach is validated against OLSR driven UAV assisted ground networks. The proposed approach is compared against software-defined UAVs (U-S) and UAVs as base stations (U-B) inspired by the configurations and settings in [28,29] for performance and efficiency analysis. The scalability of the proposed technique is verified by varying aerial node deployment and data rates. The section is further discussed in four parts: Simulation settings, Accuracy and correctness, Performance and efficiency analysis, Scalability test.

### 5.1. Simulation Settings

Simulation results are dependent on data rates and packet size. The complete simulation settings are provided in Table 1. The following parameters are used to test the efficiency of the approach:

1. *Throughput*: Throughput is the measure of the amount of data successfully transmitted between source and destination over a transmission media. The comparative analysis is performed by equating maximum data rates in UAV-assisted ground networks $Th_{maxw}$.
2. *Delay*: Delay is a unit time measurement of end-point to end-point communication considering network bottlenecks and unavailability of transmission media. Latency is the cumulative measurement of propagation and serialization delays. Although there exists a subtle distinction between latency and delay, but the proposed approach latency and delay are used interchangeably as the delay is defined as a cumulative entity comprising of router traversal latency, queuing delay, transmission delay, hop count, propagation delay, and connection delay.
3. *Jitter*: Jitter quantifies delay-sensitive dynamic network behavior. The proposed approach considers jitter as the variation in delay.
4. *Packet Delivery Ratio (PDR)*: Packet delivery ratio is the ratio of packets transmitted by the sender to the actual number of packets received at the destination node.
5. *Packet Loss*: Packet loss accounts for the number of packets lost in transmission. The proposed approach ascertains it as a proportion between unsuccessful transmissions and the actual number of transmission over the transmission media. Packet loss can occur with UAV being out of range, congestion, frequent broadcasts from ground nodes, dense ground sections, or the overall amount of data being transmitted.

### 5.2. Accuracy and Correctness

**Figure 7:**
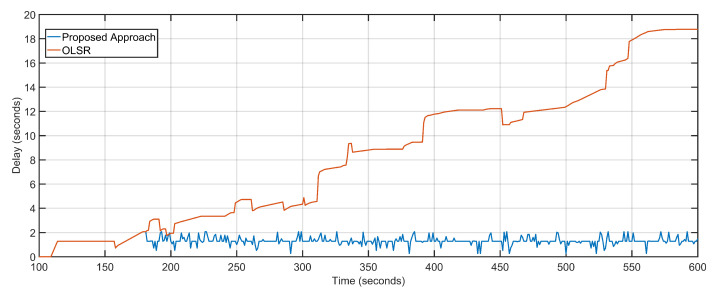
Delay: Proposed approach vs. OLSR configuration.

Multi-UAV-assisted ground networks are multi-hop network configurations where aerial nodes act as transceivers or base stations. The correctness of the proposed approach is demonstrated by running the multi-UAV-assisted ground network configuration in conjunction with the proposed approach. The underlying ground configuration uses OLSR for multi-hop data routing. The largest contributor to network latency in a multi-hop network is the number of hops between the communicating nodes and the actual geographical distance between the nodes. Bottleneck congestions occurring as a result of unequal data transmission capacity of links and devices also contribute substantially to the overall network delays. Multi-hop networks also suffer from suboptimal routing conditions by making suboptimal paths to the destination. Suboptimal path selection increases the overall data arrival intervals. The proposed approach provides solutions to the multi-hop delays and bottleneck congestions by facilitating additional resources in form of UAV re-purposing. The choice of more than one aerial node reduces the suboptimal routing paths as well. Figure 7 reflects the decrease in overall delay as the proposed approach features an average delay of 1.3 s compared to an 8.6-s delay of OLSR configuration.

**Figure 8:**
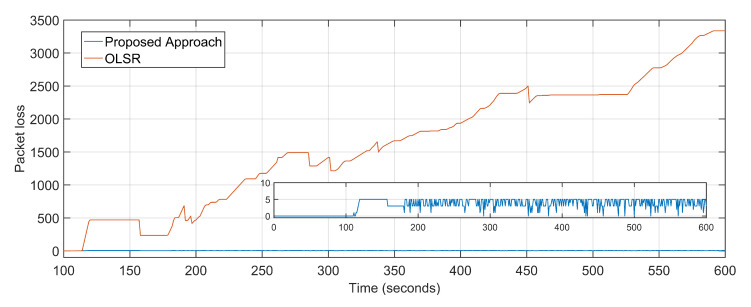
Packet Loss: Proposed approach vs. OLSR configuration.

**Figure 9:**
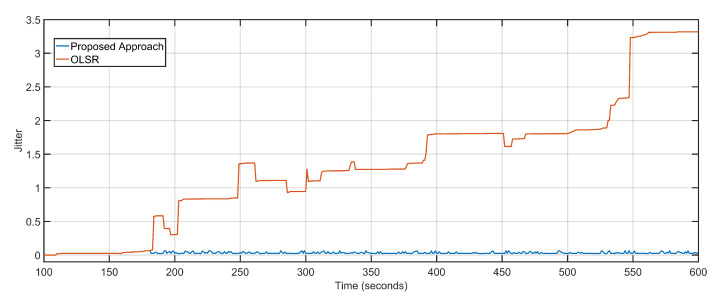
Jitter: Proposed approach vs. OLSR configuration.

The average packet loss of the proposed approach is 4 as compared to the staggering 1672 of the OLSR configuration (Figure 8). The average jitter, also considered as the variation in delay over time, of the proposed approach is 0.03 and that of the OLSR configuration is 1.4 (Figure 9). The proposed approach minimizes the distance as well as the number of hops between the source and destination, resulting in fewer packets becoming lost in transition. Random non-optimized mobility of the aerial nodes adds massively to the packet drop statistics. Packet loss is also directly equated to network congestions and overloaded links and devices. Packets are dropped in volumes if the end device or transmission channel cannot cope with the transmission rates. The unprecedented advantage of the proposed approach towards delay minimization contributes to the advantage when it comes to packet loss rates.

**Figure 10:**
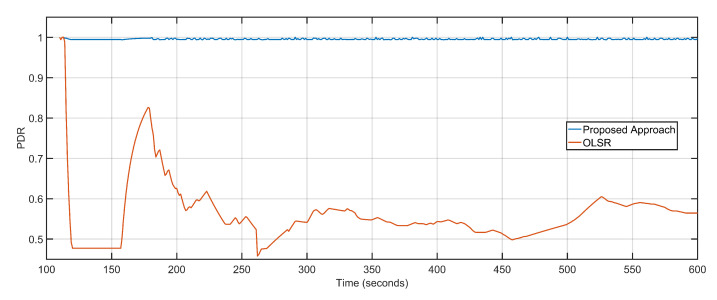
Packet delivery ratio: Proposed approach vs. OLSR configuration.

**Figure 11:**
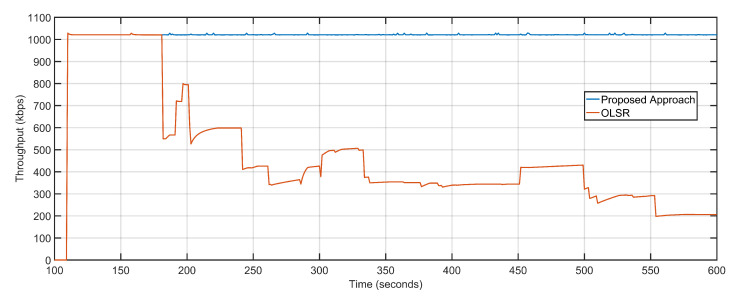
Throughput: Proposed approach vs. OLSR configuration.

The proposed approach is directed towards throughput maximization by minimizing the overall network delays and packet loss. The minimal packet loss rate converts to a high packet delivery ratio. The PDR of the proposed approach is 0.99 as compared to the OLSR configuration’s 0.55 (Figure 10). Minimized delay of 1.30 s, less average packet loss of 4, and high PDR of 0.99 result in maximized throughput values. The throughput of the proposed approach is 1021.25 Kbps, as compared to 480.42 Kbps of OLSR network configuration (Figure 11). Table 2 details the comparative analysis between the proposed approach and OLSR ground network configuration.

### 5.3. Performance and Efficiency Analysis

The performance and efficiency of the proposed approach are evaluated against software-defined UAVs (U-S) and UAVs as base stations (U-B). The three approaches are tested on the same testbed with 50 UAVs. The packet size is 1460 bytes. The data rate is 1 Mbps with a constant bit rate and variable bursts.

**Figure 12:**
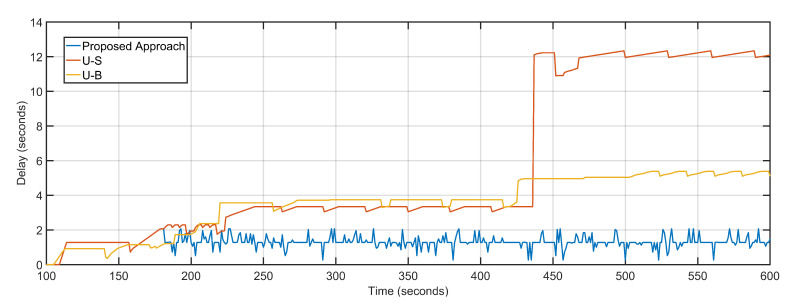
Delay: Proposed Approach, U-S, U-B.

The proposed approach features an average delay of 1.30 s as compared to 5.8 s and 3.67 s of U-S and U-B respectively (Figure 12). The proposed approach dynamically tracks the aerial topology for bottleneck congestions by keeping track of individual delays and packet loss of the aerial nodes, as well as the sector, where nodes are deployed. Both U-S and U-B feature geographical positioning of nodes with respect to the load characteristics of the underlying geography. U-B has a slight edge over U-S because after fixing the UAV positions U-B keeps them static. Thus, featuring fewer average delays in the regions where UAVs are deployed which bring down the overall average delay.

**Figure 13:**
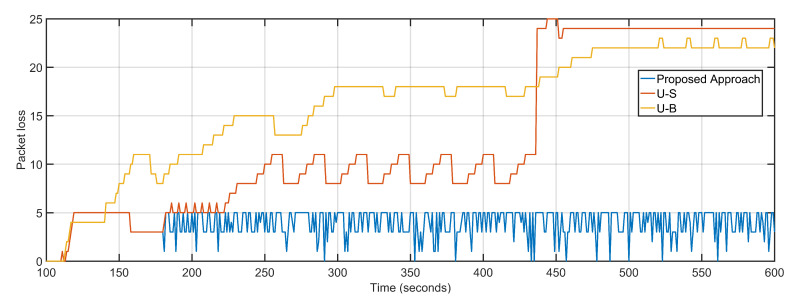
Packet Loss: Proposed Approach, U-S, U-B.

**Figure 14:**
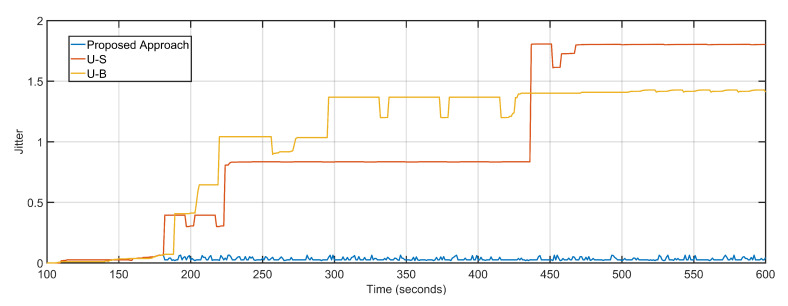
Jitter: Proposed Approach, U-S, U-B.

The average delay and packet loss of the system depends massively on the distance and number of hops over which data is transferred. The proposed approach not only re-purposes the UAV towards over-burdened nodes but also takes distance and hop count into account by measuring the 3D distance in terms of signal propagation. U-S and U-B have average packet loss of 13 and 16 respectively as compared to four of the proposed approaches (Figure 13). The low average packet drop is derived from the predictive dynamic tracking of the proposed approach. The packet loss of U-B is higher than U-S despite boasting lesser delays is because after keeping UAV positioning static, the remaining sectors experience a steep decline in packet delivery. The proposed approach, U-S, and U-B feature average jitter of 0.03, 0.99, and 1.04, respectively (Figure 14).

**Figure 15:**
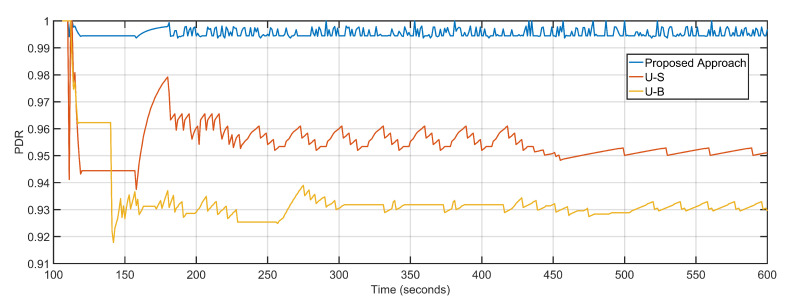
Packet delivery ratio: Proposed Approach, U-S, U-B.

**Figure 16:**
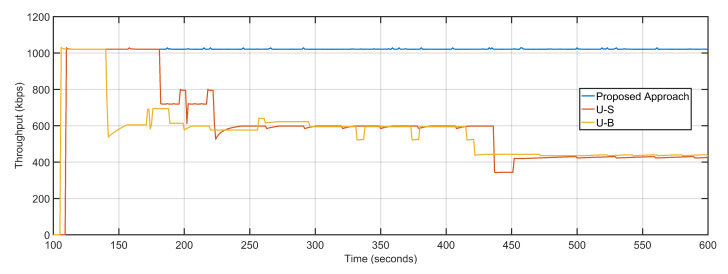
Throughput: Proposed Approach, U-S, U-B.

Less delay and near-optimal packet loss result in maximized PDR and throughput of the proposed approach are obtained compared to its counterparts. The PDR values of the proposed approach, U-S and U-B are 0.99, 0.95 and 0.93 respectively (Figure 15). The efficient re-purposing and dynamic tracking of nodes and overall topology result in maximum throughput gains. The average throughputs of the proposed approach, U-S and U-B are 1021.25 Kbps, 609.81 Kbps, and 569.94 Kbps, respectively (Figure 16). Table 3 details the comparative analysis between the three approaches.

### 5.4. Scalability Test

The scalability of the proposed approach is tested by varying the number of aerial nodes and data rates. Simulations are performed by varying the data rate 1–4 Mbps. Another test was performed by keeping the data rate constant to 1 Mbps and varying the deployed UAVs between 50–100. The packet size is 1460 bytes with a constant bit rate and variable bursts.

**Figure 17:**
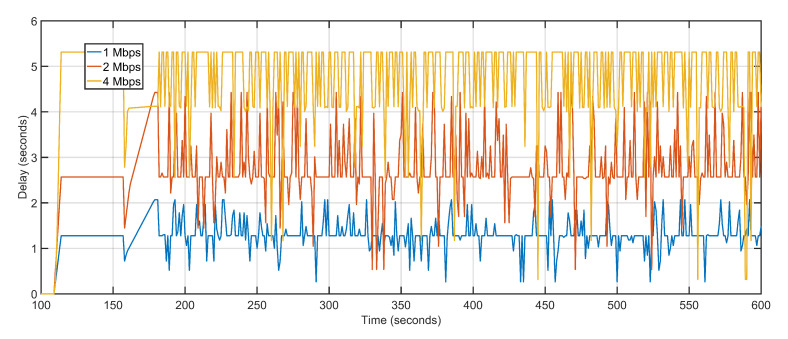
Delay: 1 Mbps, 2 Mbps, 4 Mbps respectively.

**Figure 18:**
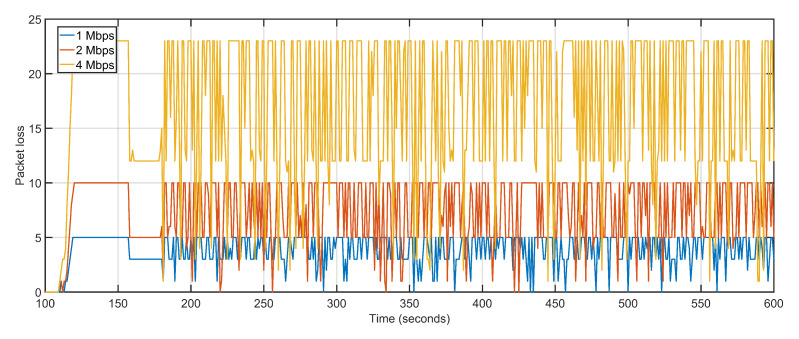
Packet Loss: 1 Mbps, 2 Mbps, 4 Mbps respectively.

**Figure 19:**
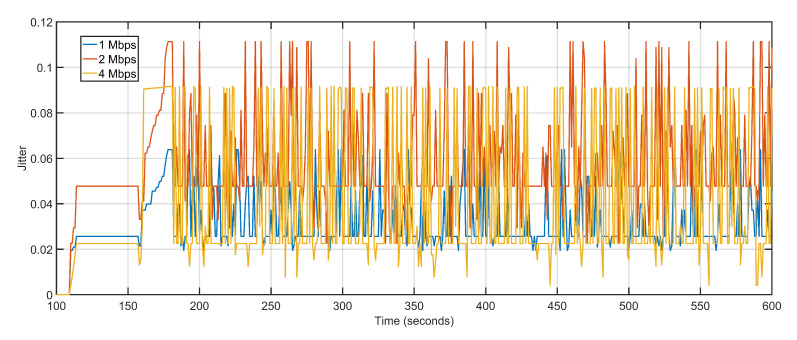
Jitter: 1 Mbps, 2 Mbps, 4 Mbps respectively.

Delay and packet loss increases slightly when data rates are gradually incremented. Increasing data rate but keeping the number of UAVs constant reverses the gains of higher data rates when it comes to delay and packet loss. The transmission capacity of the ground and aerial devices increases but the number of UAVs available for reception and re-purposing remains the same. The higher data generation with lesser available sinks pushes back the networks towards multi-hoping and sub-optimal path selections. This abrupt behavior is effectively managed by the proposed approach which sees only a slight variation in delay values. The average delay values at 1 Mbps, 2 Mbps, and 4 Mbps are 1.30 s, 2.78 s, and 4.70 s, respectively (Figure 17). With increasing data rates, the packet drop at the interface queue is affected. High data rates cause congestion and overflow in the forwarding node resulting in packet drops. The average packet drop at 1 Mbps, 2 Mbps, and 4 Mbps are 4, 8, and 17, respectively (Figure 18). The average jitter at 1 Mbps, 2 Mbps, and 4 Mbps are 0.03, 0.05, and 0.04, respectively (Figure 19).

**Figure 20:**
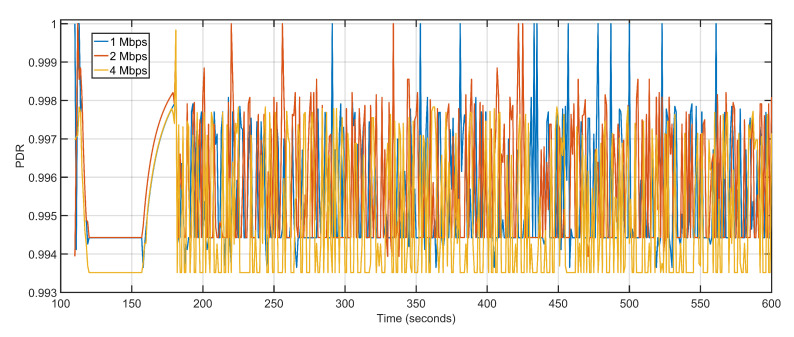
Packet delivery ratio: 1 Mbps, 2 Mbps, 4 Mbps respectively.

**Figure 21:**
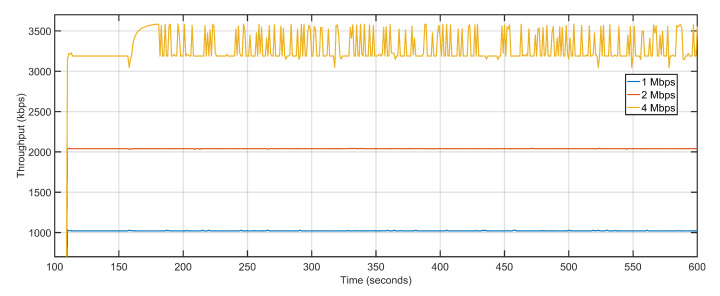
Throughput: 1 Mbps, 2 Mbps, 4 Mbps respectively.

The microscopic variations in delay and packet loss rates keep the average PDR levels constant (Figure 20). Throughput is affected marginally with data rate being increased from 1 Mbps to 2 Mbps, with values shifting from 1021.25 Kbps to 2039.26 Kbps. The only noticeable difference in throughput arrives when the data rate is further doubled from 2 Mbps to 4 Mbps and the throughput levels rise from 2039.26 Kbps to 3284.56 Kbps (Figure 21). Table 4 details the performance of the proposed approach with varying data rates. Simulation tests over iteratively increasing data rates suggest that an efficient compromise between the available number of nodes and data rate is required to achieve consistent performance levels.

**Figure 22:**
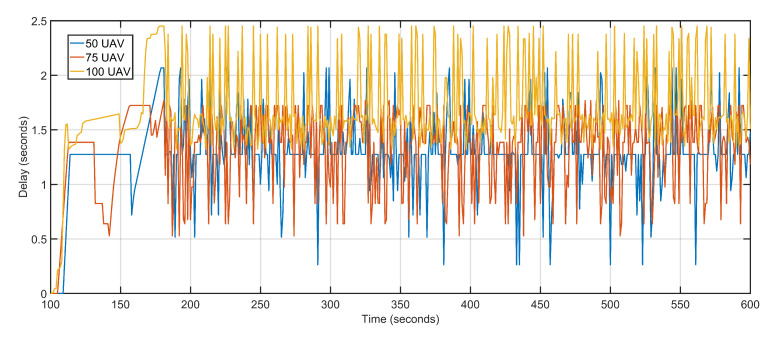
Delay. 50 UAVs, 75 UAVs, 100 UAVs respectively.

**Figure 23:**
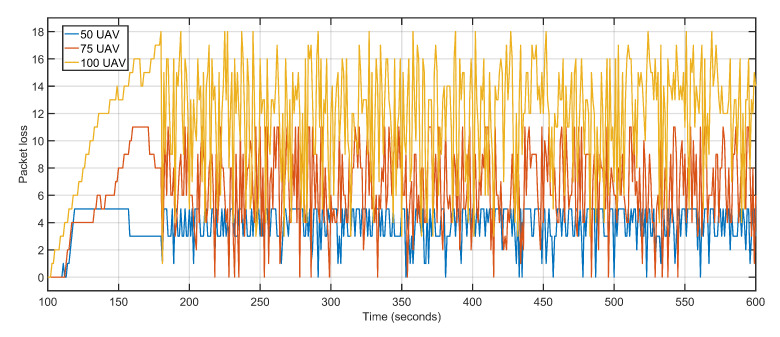
Packet Loss. 50 UAVs, 75 UAVs, 100 UAVs respectively.

**Figure 24:**
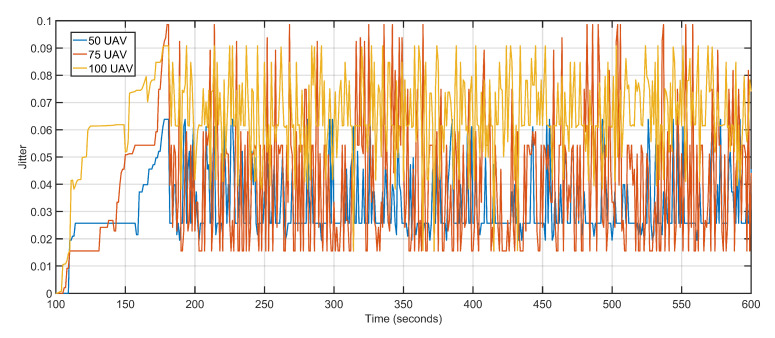
Jitter. 50 UAVs, 75 UAVs, 100 UAVs respectively.

The second scalability run is performed by keeping the data rate constant at 1 Mbps and varying the number of aerial nodes between 50, 75, and 100. Iteratively increasing the number of nodes witnesses a gradual but slow rise in delay and packet loss. The increased delay and packet loss are a result of more devices contending for the same transmission channels resulting in congestion and backoffs. An aerial node anticipates congestion in the network resulting from more and more nodes competing for the same channel. The node initializes its backoff counter and waits for a random amount of time before retransmitting. When the transmission is re-attempted, congestion is detected again, resulting in an incremented backoff value. The congestion persists as a result of increasing the number of aerial nodes but not maintaining sufficient data rates. The average delay values of 50 UAVs, 75 UAVs and 100 UAVs are 1.30, 1.34 and 1.71 respectively (Figure 22). The average packet losses after deploying 50 UAVs, 75 UAVs, and 100 UAVs are 4, 7, and 12 respectively (Figure 23). The average jitters at 50 UAVs, 75 UAVs and 100 UAVs are 0.03, 0.04 and 0.06 respectively (Figure 24).

**Figure 25:**
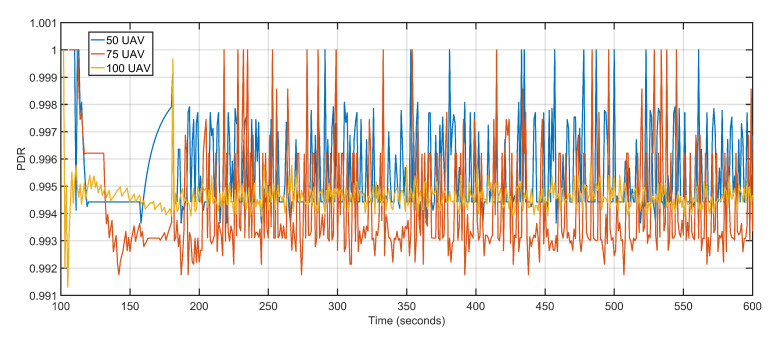
Packet delivery ratio. 50 UAVs, 75 UAVs, 100 UAVs respectively.

**Figure 26:**
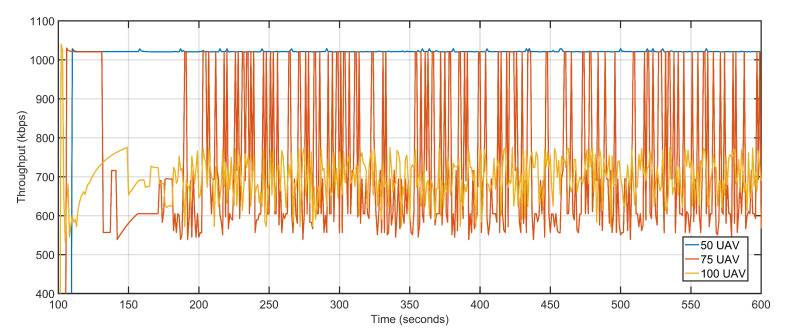
Throughput. 50 UAVs, 75 UAVs, 100 UAVs respectively.

Once again, the microscopic variations in delay and packet loss rates have less impact on average PDR (Figure 25). The throughput on the other hand witnessed a steep decline as the number of aerial nodes increase. One of the factors behind the decline is that more and more nodes contest for channel access causing congestion and nodes to back off, the other reason being the congestion and backoffs activate the proposed approach towards re-purposing UAVs. The high re-purposing results in high mobility of the aerial nodes, which in turn affects the overall throughput. The average throughputs at 50 UAVs, 75 UAVs, and 100 UAVs are 1021.25 Kbps, 728.57 Kbps, and 696.34 Kbps, respectively (Figure 26). Table 5 details the contrast in the execution of the proposed technique when the numbers of aerial nodes are varied.

## 6. Conclusions

In this article, a UAV re-purposing-based approach for throughput maximization, delay, and packet loss minimization is presented. The proposed approach employs GNN-based dynamic learning and prediction mechanism to re-purpose available UAVs towards the congested and over-burdened sectors of the topology. The state monitoring neural network architecture makes the approach more reactive and aggressive while at the same time channel prorogation based distancing makes the solution more efficient. The proposed approach is compared against the OLSR-driven UAV-assisted ground network model to demonstrate the correctness. The proposed UAV re-purposing technique tremendously outperforms the classical approach with notable gains in throughput and packet delivery ratio while at the same minimizing the losses. Moreover, a comparative study of the proposed approach is provided by comparing it against U-S and U-B. The approach establishes its supremacy by demonstrating considerable gains over U-S and U-B when compared on the merits of throughput while at the same time achieves lesser delays and packet loss as compared to the two models. The scalability analysis of the proposed approach demonstrates the effectiveness of the approach under adverse scenarios. Scalability test also establishes another important general network characteristic that the data rate and the number of devices must work together under guided compromise to archive significant gains.

Future works include developing a software defined network (SDN) controller for trajectory optimization. Mid-flight routine modification and network independence facilitated by the SDN controller will make the framework more robust and flexible while boosting scalability at the same time. The interface independent transition supported by SDN will make the proposed approach more reactive and fast to abrupt network changes. The framework can be modified to incorporate energy levels of aerial and ground nodes as part of the feature vector to avoid transmission black holes. A separate algorithm can be developed for monitoring parameter changes over time and facilitate more robust parameter predictions.

The simulation results prove that an efficient compromise between the data rate and the number of nodes is required to maintain consistent system performance levels. Increasing data rates without changing the number of nodes or increasing the number of nodes without improving the data rate leads to a gradual decline in system performance.

## Figures and Tables

**Table 1:** Simulation Parameters.

Simulation Settings	Values
No. of Ground Nodes	200
No. of Aerial Nodes	50–100
Ground Node Classification	Wireless Ground Nodes
Dimension	1200×1200 m${}^{2}$
Ground Communication	IEEE 802.11, Direct Sequence Spread Spectrum (DSSS) Rate 11 Mbps
Aerial-Ground Communication	Low Power Wide Area Network (LPWAN), 2 km Line of Sight Transmission
Aerial-Aerial Communication	LTE
Loss Model	Fiss Propagation Loss Model
Datagram/Segment Size	1460 bytes
Data Rate	1–4 Mbps
Data Burst	Variable
Bit Rate	Constant
Protocol	Transmission Control Protocol (TCP)/User Datagram Protocol (UDP)
Simulation	NS3 (Python Bindings)
Analysis	Python

**Table 2:** Comparison: Proposed approach vs. OLSR configuration.

Approach	Delay	Packet Loss	Jitter	PDR	Throughput
Proposed Approach	1.30	4	0.03	0.99	1021.25 Kbps
OLSR	8.55	1672	1.4	0.55	480.42 Kbps

**Table 3:** Comparison: Proposed approach vs. U-S vs. U-B.

Approach	Delay	Packet Loss	Jitter	PDR	Throughput
Proposed Approach	1.30	4	0.03	0.99	1021.25 Kbps
U-S	5.80	13	0.99	0.95	609.81 Kbps
U-B	3.67	16	1.04	0.93	569.94 Kbps

**Table 4:** Scalability Test: 1 Mbps, 2 Mbps and 4 Mbps.

Data Rate	Delay	Packet Loss	Jitter	PDR	Throughput
1 Mbps	1.30	4	0.03	0.99	1021.25 Kbps
2 Mbps	2.78	8	0.05	0.99	2039.26 Kbps
4 Mbps	4.70	17	0.04	0.99	3284.56 Kbps

**Table 5:** Scalability Test: 50 UAVs, 75 UAVs and 100 UAVs.

No. of UAVs	Delay	Packet Loss	Jitter	PDR	Throughput
50	1.30	4	0.03	0.99	1021.25 Kbps
75	1.34	7	0.04	0.99	728.57 Kbps
100	1.71	12	0.06	0.99	696.34 Kbps
